# Honokiol and Magnolol Exert an Anti‐Inflammatory Effect by Inhibiting JAK2/STAT3/IL17 Signalling in a Rat Model of Ulcerative Colitis: A Combination of Bioinformatics and Experimental Study

**DOI:** 10.1111/jcmm.71080

**Published:** 2026-03-19

**Authors:** Zhaoxu Cai, Shaojun Lu, Jingan Chen, Xiaoshan Yang, Junlin Lu, Changwen Feng

**Affiliations:** ^1^ Department of Pharmacy The First People's Hospital of Zhaoqing Zhaoqing China; ^2^ Laboratory Animal Center Guangdong Pharmaceutical University Guangzhou China

**Keywords:** anti‐inflammation, honokiol and magnolol, IL17, JAK2/STAT3, ulcerative colitis

## Abstract

Ulcerative colitis (UC) is a chronic, inflammatory bowel disease with limited clinical treatment. Traditional Chinese medicinal ingredients honokiol and magnolol have potential anti‐inflammatory and gastrointestinal protective effects. However, their anti‐inflammatory potential has not been investigated in UC. This study hypothesized that honokiol and magnolol alleviate UC by targeting key inflammatory signalling pathways. To verify this, we established a 2,4‐dinitrobenzenesulfonic acid‐induced UC rat model and administered honokiol and magnolol orally. The results showed that the two ingredients significantly reduced the disease activity index and colonic mucosal damage index and downregulated serum levels of pro‐inflammatory factors TNF‐α, IL‐17, and CRP. Histopathological examination showed marked alleviation of colonic mucosal hyperemia, inflammatory infiltration, and ulcerative damage following honokiol and magnolol treatment. Bioinformatics analysis identified 74 UC‐related targets for honokiol and 62 for magnolol, which were enriched in inflammatory response and JAK–STAT/IL‐17 signalling pathways. A gradient boosting machine model was established to screen 16 shared hub targets, among which IL17A, JAK2, and STAT3 were highly correlated with immune cell infiltration. Molecular docking confirmed that honokiol and magnolol could stably bind to key proteins of the JAK–STAT pathway via noncovalent interactions with low binding energy. Immunohistochemistry and Western blot further verified that both ingredients significantly inhibited the activation of IL17A, JAK2, and STAT3 in the colonic tissues of UC rats. This study demonstrates that honokiol and magnolol exert anti‐inflammatory effects in UC rats by inhibiting the JAK2/STAT3/IL17 pathway, providing a mechanistic basis and potential targets for the application of traditional Chinese medicinal ingredients in UC treatment.

## 1 Introduction

Ulcerative colitis (UC) is a chronic inflammatory bowel condition characterized by mucosal ulcer damage and persistent inflammation in both the colon and rectum, often leading to typical clinical symptoms including abdominal pain, diarrhoea, as well as recurrent mucous, purulent, and bloody stools ([1]). Epidemiological data indicate that the global incidence and prevalence of UC are rising steadily; notably, disease burden remains highest in Europe and the United States, whereas incidence rates in Asia, Africa, and other developing countries remain comparatively lower [2]. Within 5 years of diagnosis, around 20% of patients with UC require hospitalisation, and 7% undergo colectomy. Moreover, UC confers a 1.7‐fold increased risk of colorectal cancer relative to the general population [3]. Although the precise aetiology of UC remains incompletely elucidated, current evidence implicates a complex interplay among dysregulated mucosal immunity, genetic susceptibility, gut microbiota dysbiosis, impaired epithelial barrier function, and environmental triggers [1, 4]. Of these, aberrant immune activation, particularly loss of tolerance to commensal microbiota and defective regulatory T‐cell responses, is a central pathogenic driver [5, 6]. Therefore, current therapeutic strategies, including antimicrobial therapy, hormone therapy, immunosuppressants, and other methods, are adopted for UC management. While effective for many patients, these agents are limited by substantial drawbacks: systemic adverse effects, prolonged treatment duration, high relapse rates, emerging antimicrobial or immunogenic resistance, and considerable economic burden [4]. Therefore, there is an urgent need for novel therapeutic agents that offer improved safety profiles, greater accessibility, and durable efficacy, particularly those capable of modulating immune homeostasis without broad immunosuppression.

Traditional Chinese medicine has advantages such as favourable safety profiles, multitarget therapeutic actions, and cost‐effectiveness [7, 8]. To promote the scientific validation and modernization of TCM, recent studies have increasingly focused on isolating and characterising monomeric bioactive ingredients and elucidating their molecular mechanisms across disease models [9, 10]. Honokiol and magnolol are the main active phenolic compounds extracted from *Magnolia officinalis* and have been confirmed to exert various pharmacological activities including anti‐inflammatory, antimicrobial, antioxidant, antidepressant, and anticancer activities (Ming‐Xin [11, 12]). The two compounds have demonstrated regulatory effects on gastrointestinal motility and antidiarrheal activity in experimental models [13, 14]. Importantly, a recent report revealed that the extract of *Magnolia officinalis* bark can prevent enterocyte death in a mouse model of colitis [15]. Moreover, honokiol and magnolol suppress proinflammatory factors such as tumour necrosis factor‐alpha (TNF‐α), interleukin (IL)‐1beta, nitric oxide (NO), and prostaglandin E2 (PGE2) [16, 17, 18]. In addition, honokiol and magnolol were reported to inhibit canonical nuclear factor kappa B (NF‐κB) signalling, thereby dampening downstream inflammatory gene expression [19]. Collectively, these findings support the therapeutic potential of honokiol and magnolol in UC. Accordingly, this study aimed to explore the efficacy and mechanism of honokiol and magnolol in experimental UC. First, we established a dinitro benzenesulphonic acid (DBNS)‐induced colitis rat model to evaluate in vivo therapeutic effects. Second, through bioinformatics methods, we found that honokiol and magnolol were enriched in inflammatory response modulation and JAK2/STAT3/IL‐17 signalling.

Increasing evidence implicates the JAK2/STAT3 signalling as a key driver of UC pathogenesis and progression [20, 21, 22, 23, 24]. For example, a traditional Chinese herbal prescription Gegen Qinlian decoction was reported to ameliorate UC symptoms and restore Th17/Treg balance in murine UC by inhibiting IL‐6‐induced JAK2/STAT3 activation [24]. Fucoxanthin, a marine carotenoid found in seaweed and microalgae, has been demonstrated to preserve colonic epithelial barrier integrity in DSS‐treated mice by blocking JAK2 and STAT3 phosphorylation [21]. Recently, baicalin, a bioactive flavonoid extracted from the roots of *Scutellaria baicalensis*, has been shown to inhibit Th17 differentiation and IL‐17 secretion in T cells by suppressing JAK2/STAT3 signalling while downregulating IL‐17RA expression in macrophages, thereby alleviating intestinal inflammation in UC [25]. Previously, honokiol and magnolol were validated to inhibit STAT3 and Akt activation in oral epithelial cells, thereby reducing CXCR3 ligand production [26]. Moreover, honokiol and magnolol have been reported to reduce IL‐17 production in DSS‐induced colitis mice and in 2,4,6‐trinitrobenzene sulfonic acid‐induced colitis in rats, respectively [27, 28]. These findings provide a robust mechanistic rationale for hypothesizing that honokiol and magnolol exert protective effects in UC by co‐ordinately targeting the JAK2/STAT3/IL‐17 signalling cascade. This study demonstrates that honokiol and magnolol exert significant therapeutic efficacy in experimental UC and identifies their putative molecular targets as promising candidates for mechanistic validation and future drug development.

## 2 Materials and Methods

### 2.1 Animals

A total of 81 Sprague–Dawley (SD) rats were purchased from BaiShiTong Biotechnology Co. Ltd. (Certificate No: SCXK (Guangdong) 2020–0051; Guangzhou, China). All rats were housed in a specific pathogen‐free facility at Guangdong Pharmaceutical University under standardised environmental conditions: temperature maintained at 23°C ± 2°C, relative humidity at 40%–70%, and a 12/12‐h light/dark cycle. All animal studies received approval from the Animal Ethics Committee at Guangdong Pharmaceutical University (Ethics No: gdpulac2021140) and adhered to the guidelines for the Care and Use of Laboratory Animals outlined by the National Institutes of Health Animal.

### 2.2 Liposome Preparation

Liposomes encapsulating honokiol were prepared using Phospholipon 90 G (10 mg/mL) as the primary phospholipid, along with honokiol or honokiol (2 mg/mL), ethanol (0.5 mL), and double‐distilled water (5 mL). The mixture was heated to 50°C and then subjected to probe sonication using a CY‐500 ultrasonic disintegrator (Optic Ivymen System, Barcelona, Spain) under controlled conditions: two cycles, each consisting of 5 s pulse‐on and 2 s pulse‐off intervals at 60% amplitude. To mitigate excessive thermal buildup during sonication, the sample vial was immersed in a room‐temperature water bath throughout the process; consequently, the final temperature of the dispersion remained approximately 52°C. For comparative characterisation purposes, blank liposomes, identical in composition but lacking honokiol, were concurrently prepared and employed as controls [29].

### 2.3 Grouping, Modelling, and Treatment

The 81 SD rats were assigned randomly into nine groups (each *n* = 9): (I) control group (Con), (II) ulcerative colitis group (UC), (III) positive‐control group (PC), (IV) negative‐control group (NC), (V) honokiol‐high dose group (HH), (VI) honokiol‐medium dose group (HM), (VII) honokiol‐low dose group (HL), (VIII) magnolol‐high dose group (MH), and (IX) magnolol‐low dose group (ML).

For UC modelling, rats in group II‐IX received enema with 0.25 mL DBNS for 30s, while those in the Con group (I) received enema with 0.25 mL physiological saline solution (Kelun Pharmaceutical Co. Ltd., Jiangxi, China) for 30 s. Before enema, rats were subjected to a 12‐h fasting period to ensure their intestines were clear. Following anaesthesia with isoflurane (Keyuan Pharmaceutical Co. Ltd., Shandong, China), a 16‐gauge gavage needle containing solution was carefully and gradually inserted to the rat's anus up to a depth of 8 cm. The rats were then inverted for 5 min to prevent the outflow of the drug, after which they were returned to their cages and allowed to recover normally.

For treatment, rats in the Con group (I) and the UC (II) group were orally administered with physiological saline (Kelun Pharmaceutical Co. Ltd). Rats in the PC group (III) were orally administered with mesalazine suspension (70 mg/kg, Mojin Biotechnology, Hebei, China), and rats in the NC group received treatment of liposomes via oral administration. Animals in the HH, HM, and HL groups (V–VII) were orally administered with 90, 30, and 10 mg/kg honokiol liposomes (McLean Biochemical Technology, Shanghai, China), while rats in the MH and ML groups (VIII–IX) were orally administered with 90‐ and 10‐mg/kg magnolol liposomes (McLean Biochemical Technology). The doses of honokiol and magnolol were selected based on previous studies conducted in rat models [30, 31, 32], which collectively indicate that doses below 100 mg/kg are well tolerated and fall within the established safety margin. Oral gavage was administered once daily at a volume of 10 mL/kg for 2 consecutive weeks.

### 2.4 Scoring

Throughout the treatment period, the disease activity index (DAI) score was calculated as the sum of three indicators: weight loss, stool consistency, and occult blood [33]. Scoring was performed according to DAI scoring criteria (Table 1) on the 1st, 2nd, 4th, 7th, and 14th days.

**TABLE 1:** DAI scoring criteria.

Score	Weight loss (%)	Stool consistency	Occult blood test
0	< 1	Normal	Negative (−)
1	1–5	Normal‐sparse stool	Weak positive (+)
2	5–10	Sparse	Positive (++)
3	10–20	Sparse stool and diarrhoea	Strong positive (+++)
4	> 20	Diarrhoea	Bloody stool

After the 2‐week treatment, rats were anaesthetised by isoflurane, and the blood was collected from femoral arteries. Following euthanasia via spinal dislocation, colonic tissues were collected from rats. The colonic tissue samples were cut along the longitudinal axis and rinsed with physiological saline solution (Kelun Pharmaceutical Co. Ltd). Next, the samples were photographed and scored according to CMDI scoring criteria shown in Table 2 [33]. Subsequently, the longitudinally dissected colon was halved. One of the halves was wrapped and fixed with 10% neutral formalin solution, while the other half was stored at 80°C.

**TABLE 2:** CMDI scoring criteria.

Score	The mucous membrane of the colon
0	No damage
1	Mild hyperaemia and oedema, smooth surface, no erosion or ulcer
2	Hyperaemia and oedema, rough mucous membrane, granular sensation, erosion or intestinal adhesion.
3	Severe hyperaemia and oedema, necrosis and ulcer formation on the surface, thickening of intestinal wall or necrosis and inflammatory polyps on the surface.
4	Severe hyperaemia and oedema, mucosal necrosis and ulcer formation, whole intestinal wall necrosis, death caused by toxic megacolon.

### 2.5 Enzyme‐Linked Immunosorbent Assay (ELISA)

The levels of IL‐17, TNF‐α, and CRP in the collected rat serum samples were detected using the corresponding ELISA kit (Meimian industrial Co. Ltd., Jiangsu, China) following the manufacturer's recommendations.

### 2.6 Histopathology

Haematoxylin and eosin staining was performed to evaluate pathological changes in colonic tissues after modelling and different treatments. In brief, colonic tissues were embedded in paraffin, sectioned (4 μm thickness), and stained with haematoxylin and eosin (SenBeiJia Biological Technology, Nanjing, China) using TR‐180 Autostainer (Taiwei Technology Industry, Hubei, China). Subsequently, all staining sections were photographed by a MOTIC scanning microscope (MOTIC1300901111071, Germany).

### 2.7 Downloads of Datasets

Five gene‐expression datasets (GSE16879, GSE87473, GSE107499, GSE87466, and GSE92415) were downloaded from the Gene Expression Omnibus database (GEO, http://www.ncbi.nlm.nih.gov/geo/). Among them, three datasets (GSE16879, GSE87473, and GSE107499) were used for differentially expressed analysis, and the other datasets (GSE87466 and GSE92415) were employed in the subsequent validation of the machine learning model.

### 2.8 Differential Expression Analysis

The differential expression analysis was performed to identify differentially expressed genes (DEGs) in samples from UC patients. Since the data had been collected by different labs, normalisation and batch‐effect correction of GSE16879, GSE87473, and GSE107499 were performed to get a merged dataset (the merge. normalise file) by “limma” and “sva” packages in R software (Version 4.4.1). Then, the merge. normalise file was used to identify DEGs using the “limma” package in R software (Version 4.4.1), and genes with |log2 FC| ≥ 0.5 and adj. *p* value < 0.05 were considered DEGs. Heatmaps and volcano plots of the DEGs were created using the “pheatmap” and “ggplot2” packages in R software (Version 4.4.1).

### 2.9 Identification of UC‐Related Targets of Honokiol and Magnolol

UC‐related genes were downloaded from GeneCards (https://www.genecards.org/). Target genes of honokiol and magnolol were collected from references [28, 34] and various target databases, namely, Traditional Chinese Medicine Systems Pharmacology Database and Analysis Platform [35], HERB [36], Pubchem [37] and Pharmmapper [38]. Then, to screen UC‐related targets of honokiol (or magnolol), the “VennDiagram” package in R was used to identify the overlapping genes among DEGs, UC‐related genes, and targets of honokiol (or magnolol).

### 2.10 Functional Enrichment Analysis

After the identification of UC‐related targets of honokiol or magnolol, their biological functions and pathway enrichment were analysed. The “clusterProfiler,” “enrichplot,” “ComplexHeatmap,” “ggplot2,” “circlize,” “RColorBrewer,” and “dplyr” packages in R were applied to analyse gene ontology (GO) function, including cellular component, molecular function, and biological process. The “clusterProfiler,” “enrichplot,” and “ggplot2” packages in R were applied to identify Kyoto Encyclopaedia of Genes and Genomes (KEGG) pathways of UC‐related targets for honokiol or magnolol.

### 2.11 Construction and Validation of Machine‐Learning Models

Before modelling, genes in the merge.normalise file (GSE16879, GSE87473, and GSE107499) were selected as a train test which was applied in machine‐learning model construction, and those in both GSE87466 and GSE92415 were selected and normalised as test sets which were applied to verify the effectiveness and accuracy of the established model. For modelling, a series of packages (“openxlsx,” “seqinr,” “plyr,” “RColorBrewer,” and “pROC”) in R were used for predictive modelling in the train set using 113 different machine‐learning algorithms (Ridge, Stepglm, LASSO, SVM, LDA, glmBoost, PplsRglm, Random Forest, GBM, etc.), and for model validation in the test set via both ROC curves and confusion matrix methods.

### 2.12 Identification of Hub UC‐Related Targets of Honokiol and Magnolol

The model genes in the best algorithms of machine‐learning models were considered the most promising gene set to further screen hub UC‐related targets of honokiol and magnolol. Protein–protein interaction (PPI) networks of UC‐related targets in the best model genes set were constructed using the STRING database (https://string‐db.org/). The organism species was set as “ *homo sapiens,* ” with the correlation degree set as ≥ 0.40. Then, hub UC‐related targets of honokiol or magnolol in PPI networks were identified by the Cytohubba module in Cytoscape 3.8.2 software and intersected to obtain the shared hub UC‐treated targets using the “VennDiagram” package in R.

### 2.13 Analysis of Immune Infiltration, GSEA Pathway, and Molecular Docking

Analyses of immune infiltration analysis, GSEA pathway, and molecular docking were performed to evaluate the importance of hub UC‐treated targets in the immune microenvironment, signalling pathways, and drug efficacy.

For immune infiltration analysis, the CIBERSORT algorithm based on gene expression was used to assess the relative abundance and differences in the infiltration of 22 immune cells in both the control group (normal samples) and treatment group (UC samples). The “corrplot” and “vioplot” packages in R were used to visualise the results. Following the revelation of immune cell infiltration, the correlation between levels of infiltrating immune cells and the expression of the shared hub UC‐treated targets was investigated via “limma,” “reshape2,” “ggpubr,” and “ggExtra” packages in R.

For GSEA, the correlation between status (activation/inhibition) of KEGG pathways and the expression of the shared hub UC‐treated targets was analysed based on “c2.cp.kegg.Hs.symbols.gmt” file via “limma,” “clusterProfiler,” and “enrichplot” packages in R.

To clarify the binding patterns of honokiol and magnolol with hub targets, molecular docking analysis was conducted. The 3D structures of IL17A (PDB ID: 7AMA), JAK2 (PDB ID: 8BPV), and STAT3 (PDB ID: 6NJS) were retrieved from the Protein Data Bank (PDB, http://www1.rcsb.org). The selection of these structures was based on the following criteria: high resolution (< 3.0 Å), completeness of the relevant domains, and the presence of a native or well‐characterised inhibitor in the co‐crystal structure, which facilitates the identification of the binding site for docking. The 3D structures of the small‐molecule ligands (honokiol and magnolol) were obtained from the PubChem database (http://pubchem.ncbi.nlm.nih.gov). After dehydration and ligand operations were removed from the protein receptor, the grid coordinates of protein pocket were generated by AutodockTools software. Molecular docking between the receptor and ligand was performed by AutoDock Vina 1.1.2 software. Finally, the interaction mechanism was presented using Discovery studio software.

### 2.14 Immunohistochemistry

Immunohistochemistry was performed to measure the expression of hub UC‐treated targets of honokiol and magnolol (IL17, JAK2, and STAT3) in rat colonic tissues in response to UC, honokiol, and magnolol treatment. Colonic tissues were embedded in paraffin and sectioned into 4 μm thickness. The sections were then blocked with serum and incubated overnight at 4°C using primary antibodies against IL‐17 (1:50 dilution), JAK2 (1:50 dilution), and STAT3 (1:50 dilution). Next, the tissues were incubated with secondary antibodies of goat anti‐mouse IgG or goat anti‐rabbit IgG (1:100 dilution) for 1 h at room temperature, followed by staining with 3,3′‐diaminobenzidine tetrahydrochloride. All stained sections were photographed by a MOTIC scanning microscope (MOTIC1300901111071, Germany). Finally, ImageJ software (Version 1.49, National Institutes of Health, Bethesda, USA) was used to quantitate the positive staining ratio of IL17, JAK2, or STAT3.

### 2.15 Western Blot Analysis

Western blot was performed to determine protein levels of IL17 and STAT3 in rat colonic tissues in each group. Colonic tissues were subjected to protein extraction using a total protein extraction reagent (Gambling Biotechnology, Shandong, China). The lysates were centrifuged at 12,000 × g for 30 min at 4°C, and the supernatant was then transferred to a pre‐chilled centrifuge tube. The protein concentration was measured using a BCA protein assay kit (#K3000, Gambling Biotechnology, Shanghai, China). Proteins were separated via SDS‐PAGE and transferred to PVDF membranes (IPVH00010, Millipore, MA, USA). The membranes were blocked with a solution of 5% skimmed milk powder at 25°C for 1 h, washed with TTBS, and subjected to overnight incubation at 4°C with primary antibodies diluted to 1:1000 (see Table 3). After that, secondary antibodies including HRP‐conjugated AffiniPure goat anti‐mouse IgG or goat anti‐rabbit IgG (1:1000 dilution; Wuhan Doctoral Biotechnology, Hubei, China) were applied at 25°C for 1 h. All blots were detected using an Automatic Gel Imaging Analyser (JS‐680A, Bilang Instrument Co. Ltd., Shanghai, China) and analysed with ImageJ software.

**TABLE 3:** Primary antibodies used in Western blot analysis.

Name of antibody	Catalogue number	Source
IL17	RT1326	Huaan Biotechnology, Hangzhou, China
STAT3	21,046	SAB Biotherapeutics, Nanjing, China
JAK2	GB11325	Servicebio, Hubei, China
GAPDH	KC‐5G4	Kangcheng Bioengineering, Shanghai, China

### 2.16 Statistical Analysis

All data in this study are presented as means ± standard deviation (SD). The statistical significance was analysed using one‐way analysis of variance followed by Tukey's *post hoc* analysis with the SPSS 27 software. *P* values less than 0.05 were considered statistically significant.

## 3 Results

### 3.1 Honokiol and Magnolol Play a Significant Anti‐Inflammatory Role in the UC Rat Model

**FIGURE 1:**
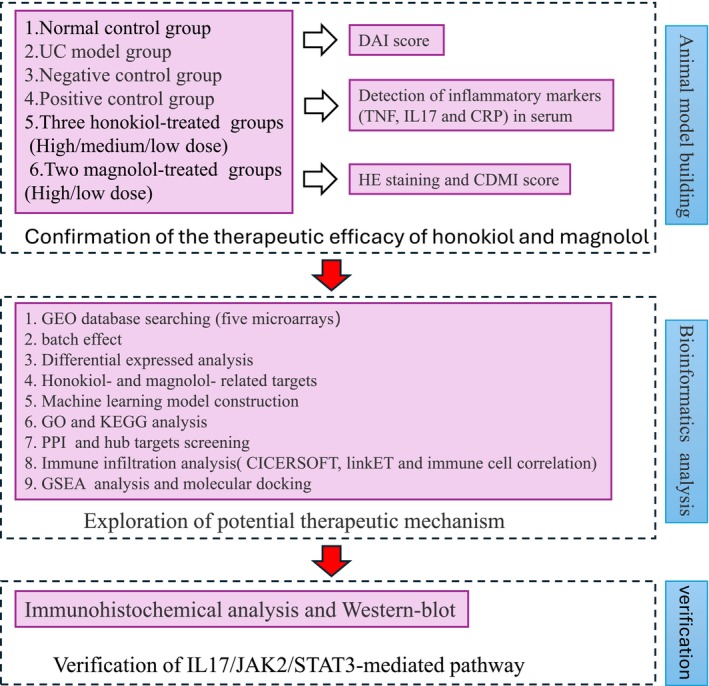
Research flowchart.

**FIGURE 2:**
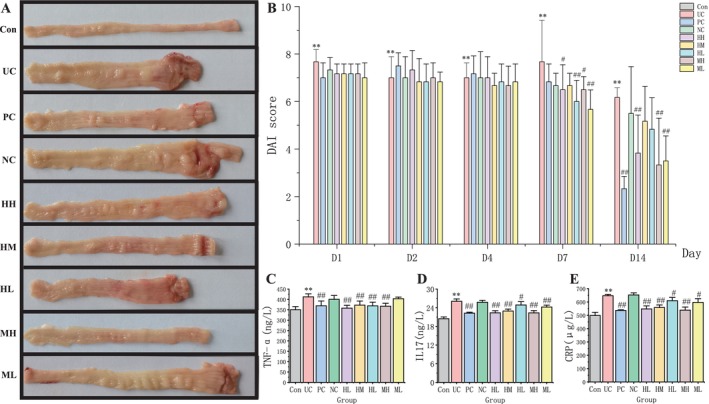
The therapeutic effects of honokiol and magnolol in UC rats. (A) Colonic anatomy of each group. (B) Disease activity index (DAI) score of each group. (C, D, and E) TNF‐α, IL17, and CRP detection in rat serum samples of each group. Data were mean ± SD. *p < 0.05 and **p < 0.01 vs. the Con group, ^#^ *p* < 0.05 and ^##^ *p* < 0.01 vs. the UC group. **Con**: Control. **UC**: Ulcerative colitis. **PC**: Positive control. **NC**: Negative control. **HH**: Honokiol‐high dose. **HM**: Honokiol‐medium dose. **HL**: Honokiol‐low dose. **MH**: Magnolol‐high dose. **ML**: Magnolol‐low dose.

**FIGURE 3:**
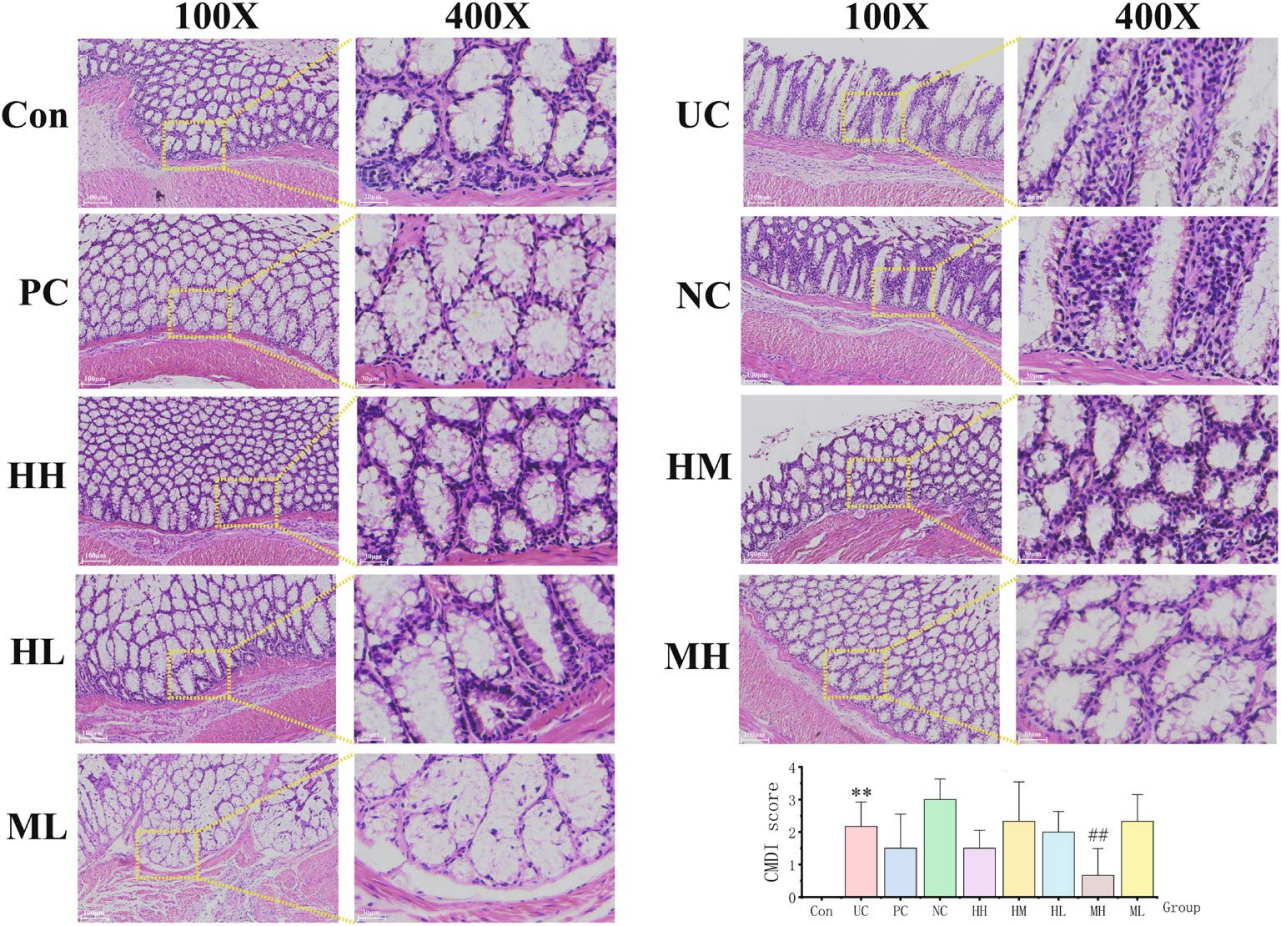
Pathological analysis and CDMI score. Haematoxylin and eosin (HE) staining was performed to analyse colonic structure of rats in each group. Data were represented as mean ± SD. *p < 0.05 and **p < 0.01 vs. the Con group, ^#^ *p* < 0.05 and ^##^ *p* < 0.01 vs. the UC group. **Con**: Control. **UC**: Ulcerative colitis. **PC**: Positive control. **NC**: Negative control. **HH**: Honokiol‐high dose. **HM**: Honokiol‐medium dose. **HL**: Honokiol‐low dose. **MH**: Magnolol‐high dose. **ML**: Magnolol‐low dose. **CDMI**: Colonic mucosal damage index.

A flow chart was used to display experimental design, drug administration, bioinformatics analysis, and following verification in this study are shown in Figure 1. After modelling and oral administration of honokiol and magnolol, the outcome of intestinal inflammation of UC rats was assessed by macroscopic, biochemical, and histological analyses (Figures 2 and 3). Compared with the control group, macroscopic inspection of the colon in the UC and NC groups showed severe colonic mucosal damage including obvious hyperaemia, crypt destruction, bowel wall thickening, ulceration, and necrosis of the mucosa. All the changes were improved in the PC, HH, HM, HL, MH, and ML groups (Figure 2A), suggesting that the positive drug mesalazine and various doses of honokiol and magnolol significantly improved DBNS‐induced colonic mucosal injury. In addition, DAI score displayed a continuously significant high value in the UC group within 14 days (*p* < 0.01 vs. control) (Figure 2B). The UC‐induced high DAI score was significantly reduced in the PC, HH, HM, HL, MH, and ML groups on Day 7 (*p* < 0.05 or *p* < 0.01 vs. UC) and in the PC, HH, MH, and ML groups on Day 14 (*p* < 0.05 vs. UC). Figure 2C–2E revealed that serum TNF‐α, IL‐17, and CRP levels in the UC group were significantly increased (*p* < 0.01 vs. control). The increased serum TNF‐α levels mediated by UC modelling were significantly reduced in the PC, HH, HM, HL, and MH groups (*p* < 0.01 vs. UC), and high serum levels of IL‐17 and CRP after modelling were significantly lessened in the PC, HH, HM, HL, MH, and ML groups (*p* < 0.01 vs. UC) (Figure 2C–2E). The results suggested that honokiol, magnolol, and the positive drug mesalazine effectively diminished serum levels of inflammatory mediators in UC model rats.

Histological results demonstrated that the colonic structure of rats in the UC and NC groups exhibited typical inflammatory ulceration in the colonic mucosa and submucosa, including goblet cell loss, massive neutrophilic infiltration, destruction of the glands, intestinal adhesion, and fibroblast proliferation (Figure 3). Moreover, honokiol and magnolol treatment markedly inhibited inflammatory cell infiltration and facilitated the recovery of the colonic structure, as shown by the alleviation of mucosal ulceration and an increase in the number of goblet cells in the HH, HM, HL, MH, and ML groups (Figure 3). Additionally, UC caused an elevation of the CDMI score (*p* < 0.01 vs. control), and the trend was significantly reversed by a high dose of magnolol (MH vs. UC *p* < 0.01) (Figure 3). The findings indicated significantly improved colonic mucosal injury.

### 3.2 Overlapping Genes Among DEGs, UC‐Related Genes and Honokiol Targets (Or Magnolol Targets)

**FIGURE 4:**
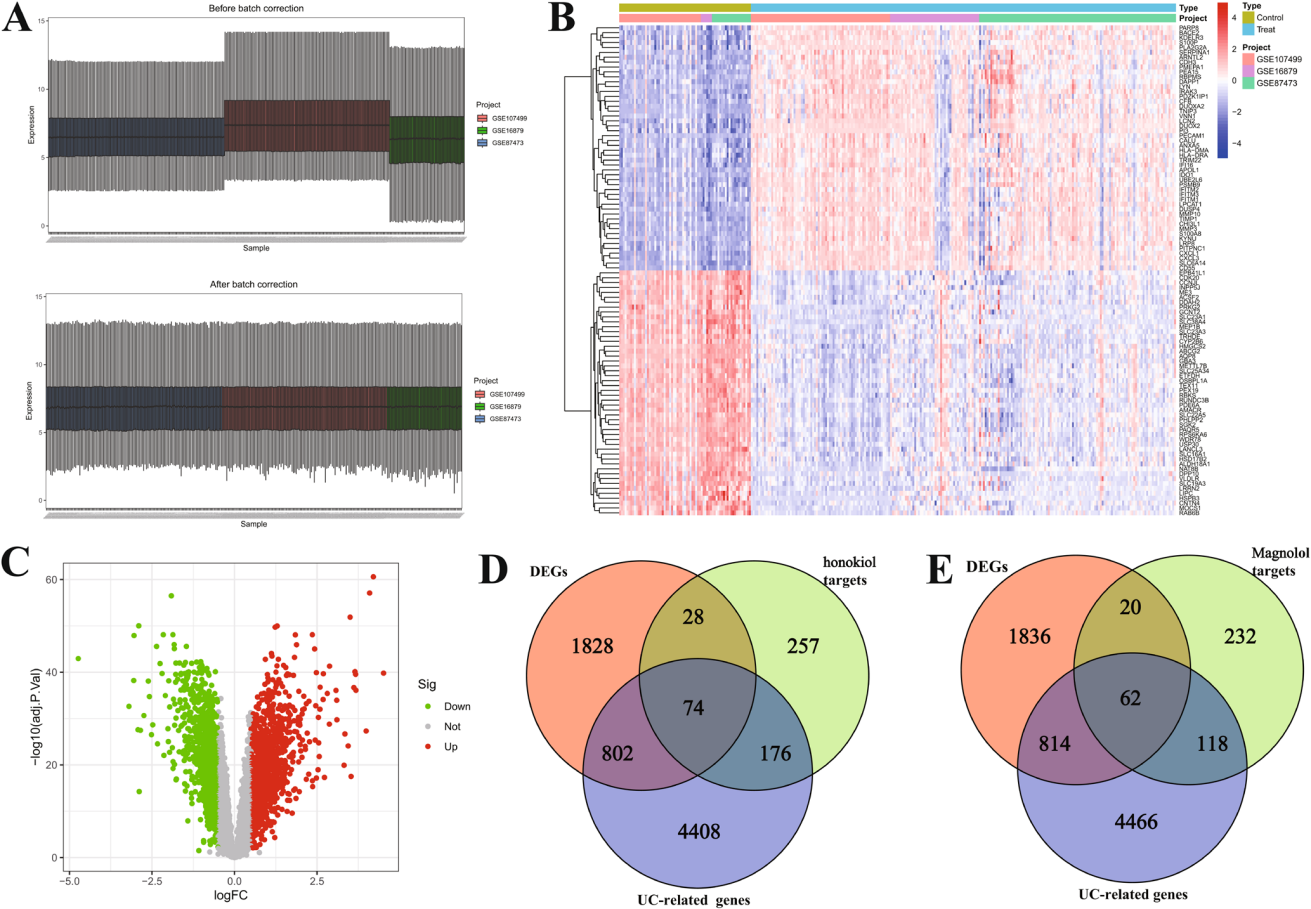
Differentially expressed analysis. (A) Boxplot of the merge normalise file (GSE16879, GSE87473, and GSE107499) prior to normalisation and post normalisation. (B and C) Clustering heatmap and volcano plot of DEGs. (D and E) Venn diagram illustrating overlapping DEGs among UC‐related genes (downloaded from GeneCards) and honokiol targets (or magnolol targets).

After successful normalisation and batch‐effect correction (Figure 4A), 2731 DEGs (1547 upregulated and 1184 downregulated genes) were identified in UC patients based on differential expression analysis from five datasets (Figure 4B,C). Venn diagrams were used to identify overlapping genes among these DEGs, UC‐related genes (from GeneCards), and targets of honokiol or magnolol. There are 74 overlapping genes among DEGs, UC‐related genes, and honokiol targets (Figure 4D) and 62 overlapping genes among DEGs, UC‐related genes, and magnolol targets (Figure 4E).

### 3.3 Functional Enrichment Analysis

**FIGURE 5:**
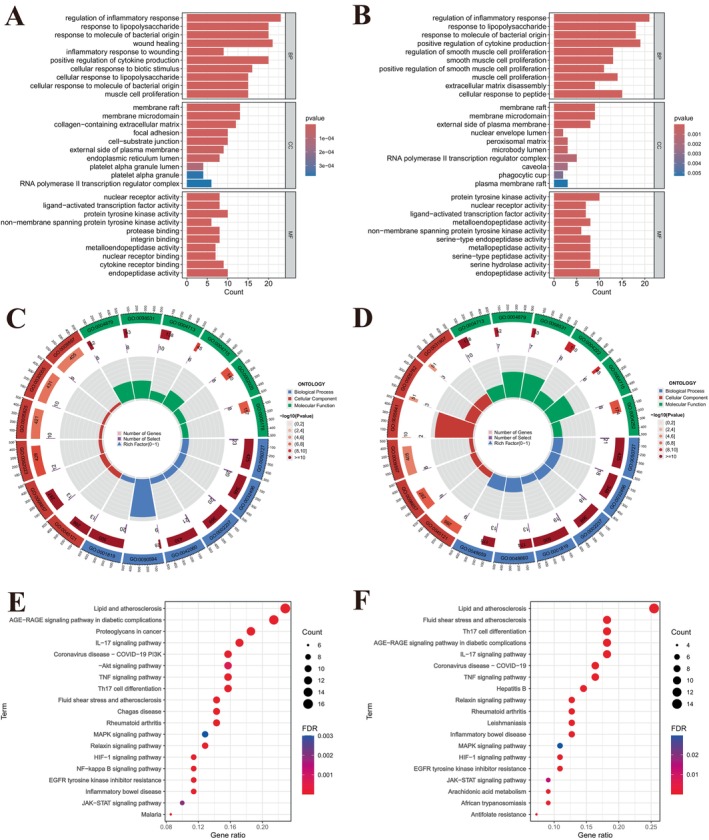
Functional enrichment analysis. (A, C) GO function analysis for UC‐related targets of honokiol is shown in a bar plot and a Circlize plot. (B, D) A bar plot and a Circlize plot were used to analyse GO functions of genes targeted by magnolol and related to UC. (E and F) Bubble plots were used to show KEGG pathway enrichment of UC‐related targets for honokiol (E) and magnolol (F).

GO function analysis showed that UC‐related targets of honokiol or magnolol were enriched in various biological processes (BP), cellular components (CC), and molecular functions (MF) (Figure 5A–5D). In terms of BP, UC‐related targets of honokiol can regulate inflammatory response, cytokine production, and muscle cell proliferation (Figure 5A,C). As to CC, membrane raft, membrane microdomain, collagen‐containing extracellular matrix, and focal adhesion are popular terms (Figure 5A,C). In the aspect of MF, UC‐related targets of honokiol possess nuclear receptor activity, protease binding ability, metalloendopeptidase activity, and cytokine receptor binding ability, etc. (Figure 5A,C). Figure 5B,D is the GO function analysis for UC‐related targets of magnolol. These genes were enriched in BP terms such as regulation of inflammatory response, muscle cell proliferation, positive regulation of cytokine production, and extracellular matrix disassembly (Figure 5B,D). Most of these genes are localised in membrane raft, membrane microdomain, nuclear envelope lumen, and peroxisomal matrix and have molecular functions such as nuclear receptor activity, serine‐type endopeptidase activity, and metalloendopeptidase activity (Figure 5B,D). According to KEGG pathway analysis, UC‐related targets of honokiol are mainly enriched in Th17 cell differentiation, IL‐17 signalling pathway, JAK–STAT signalling pathway, inflammatory bowel disease, and NF‐kappa B signalling pathway (Figure 5E). While UC‐related targets of honokiol are mainly enriched in Th17 cell differentiation, IL‐17 signalling pathway, inflammatory bowel disease, JAK–STAT signalling pathway, and TNF signalling pathway (Figure 5F).

### 3.4 Successful Establishing of the Gradient Boosting Machine (GBM) Learning Model for Honokiol Treatment

**FIGURE 6:**
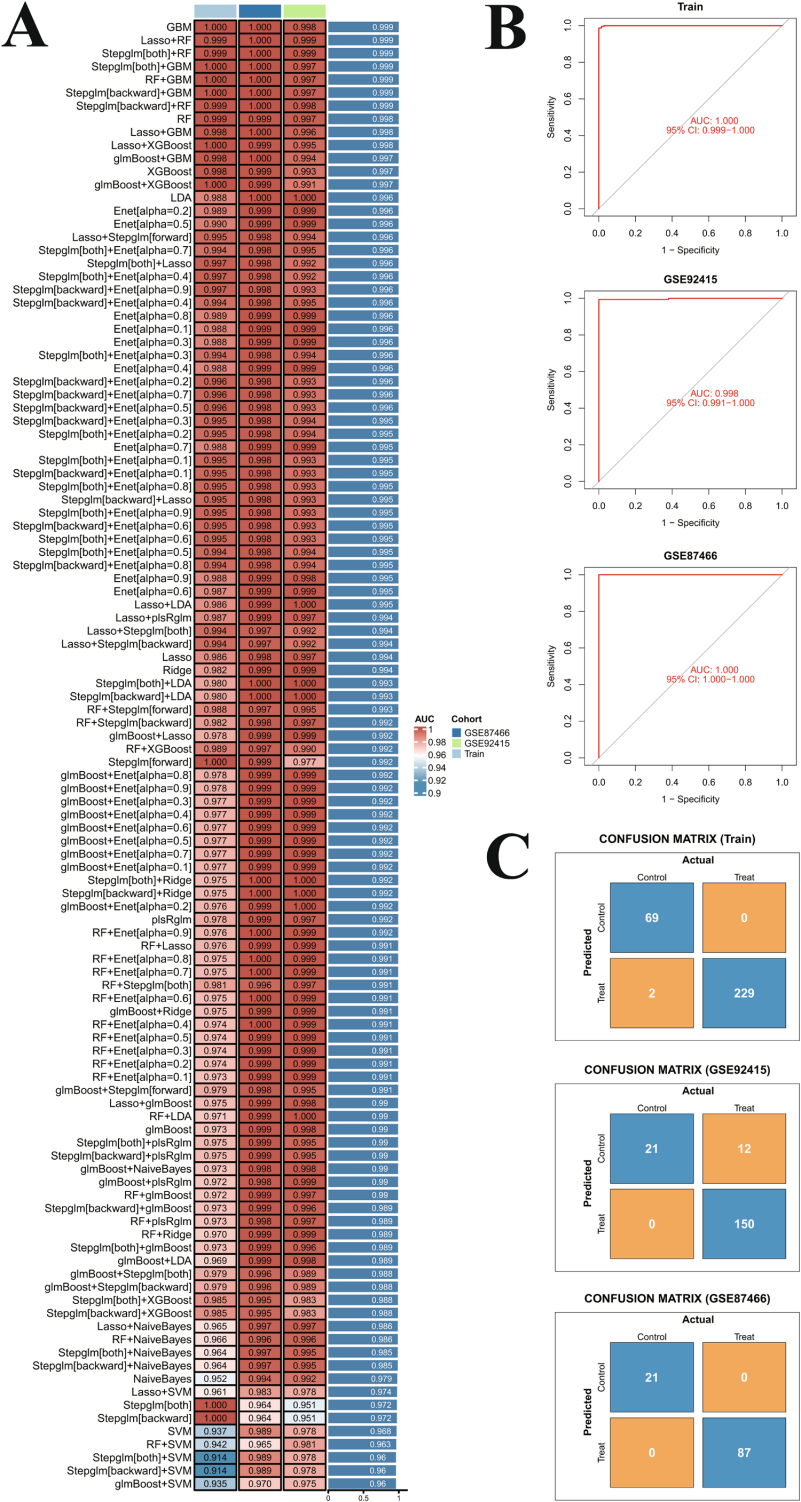
Establishment and validation of **the** machine‐learning model for honokiol treatment. (A) Clustering heatmap of 113 machine‐learning models. (B) ROC curve plots for GBM model genes in the train set, GSE87466 test set, and GSE92415 test set. (C) Confusion matrix plots for GBM model genes in the train set, GSE87466 test set, and GSE92415 test set.

After the calculations, the GBM model consisting of 74 UC‐related targets of honokiol yielded the best predictive performance among the 113 algorithms tested. The ROC values for diagnosing UC using the GBM model in train test (GSE16879, GSE87473, and GSE107499), GSE87466 test set, and GSE92415 test set were 1.000, 1.000, and 0.998, respectively (Figure 6A,B). In the confusion matrix, the accuracy of the GBM model for binary classifications, namely, control (normal) samples and treat (UC) samples, was above 60% in train test (GSE16879, GSE87473, and GSE107499), GSE87466 test set, and GSE92415 test set (Figure 6C). These results showed the GBM model was successfully built, and the 74 UC‐related targets of honokiol could be applied in the diagnosis of UC and for the further screening of hub targets in the treatment with honokiol.

### 3.5 Selection and Verification of the GBM Model for Magnolol Treatment

**FIGURE 7:**
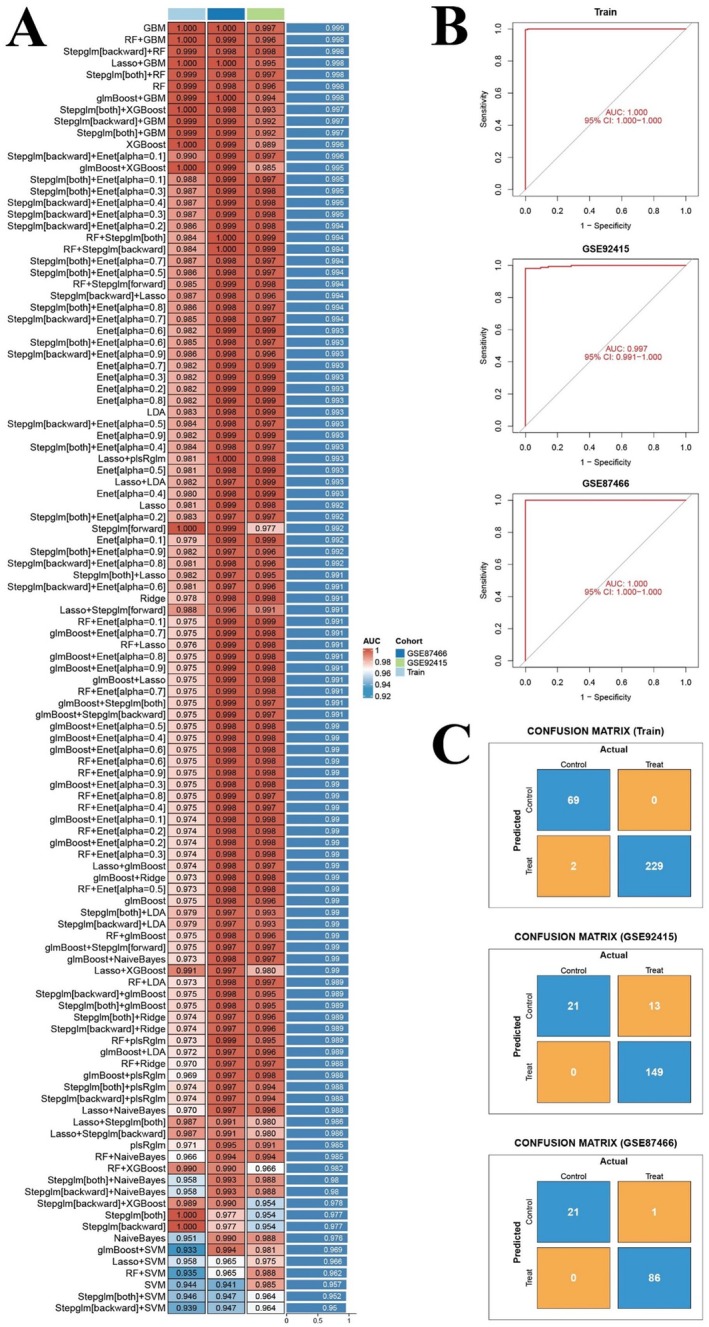
Establishment and validation of machine‐learning model for magnolol treatment. (A) Clustering heatmap of 113 machine‐learning models. (B) ROC curve plots for GBM model genes in the train set, GSE87466 test set, and GSE92415 test set. (C) Confusion matrix plots for GBM model genes in the train set, GSE87466 test set, and GSE92415 test set.

The GBM model consisting of 62 UC‐related targets of magnolol yielded the best predictive performance among the 113 algorithms tested. The ROC values for diagnosing UC using the GBM model in the train test (GSE16879, GSE87473, and GSE107499), GSE87466 test set, and GSE92415 test set were 1.000, 1.000, and 0.997, respectively (Figure 7A,B). In the confusion matrix, the accuracy of the GBM model for binary classifications, namely, control (normal) samples and treat (UC) samples, was above 60% in the train test (GSE16879, GSE87473, and GSE107499), GSE87466 test set, and GSE92415 test set (Figure 7C). These results showed the successful establishment of the GBM model, and the 62 UC‐related targets could be applied in the diagnosis of UC and for further screening of hub targets in magnolol treatment.

### 3.6 Identification of Hub UC‐Related Targets of Honokiol and Magnolol

**FIGURE 8:**
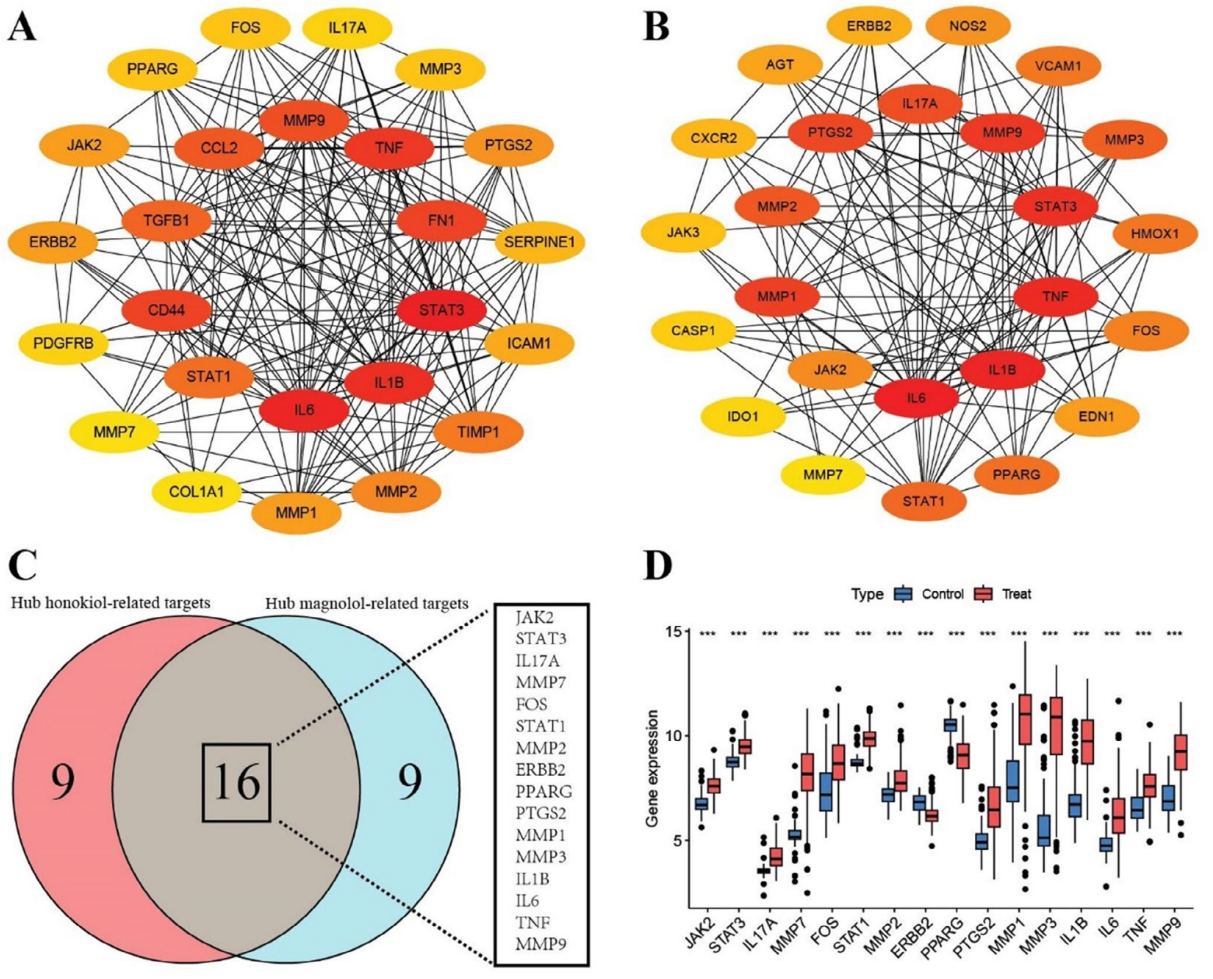
Identification of hub honokiol‐UC and magnolol‐UC targets. (A) PPI networks of hub honokiol‐UC targets. (B) PPI networks of hub honokiol‐UC targets. (C) Venn diagram illustrating the shared hub UC‐treated targets of honokiol and magnolol. (D) Gene expressions of the shared hub UC‐treated targets. ***p < 0.001.

According to the cytoHubba plugin's Degree ranking, PPI networks were used to display the top 25 hub UC‐related targets of honokiol (Figure 8A) and the first 25 hub UC‐related targets of magnolol (Figure 8B). Among the 50 genes, 16 of them were intersected (Figure 8C). The intersected genes include 2 downregulated genes (*ERBB2 and PPARG*) and 14 upregulated genes (*JAK2, STAT3, IL17A, MMP7, FOS, STAT1, MMP2, PTGS2, MMP1, MMP3, IL1B, IL6, TNF, and MMP9*) in UC samples (Figure 8D).

### 3.7 Hub Targets of Honokiol and Magnolol Affects the Immune Microenvironment in UC

**FIGURE 9:**
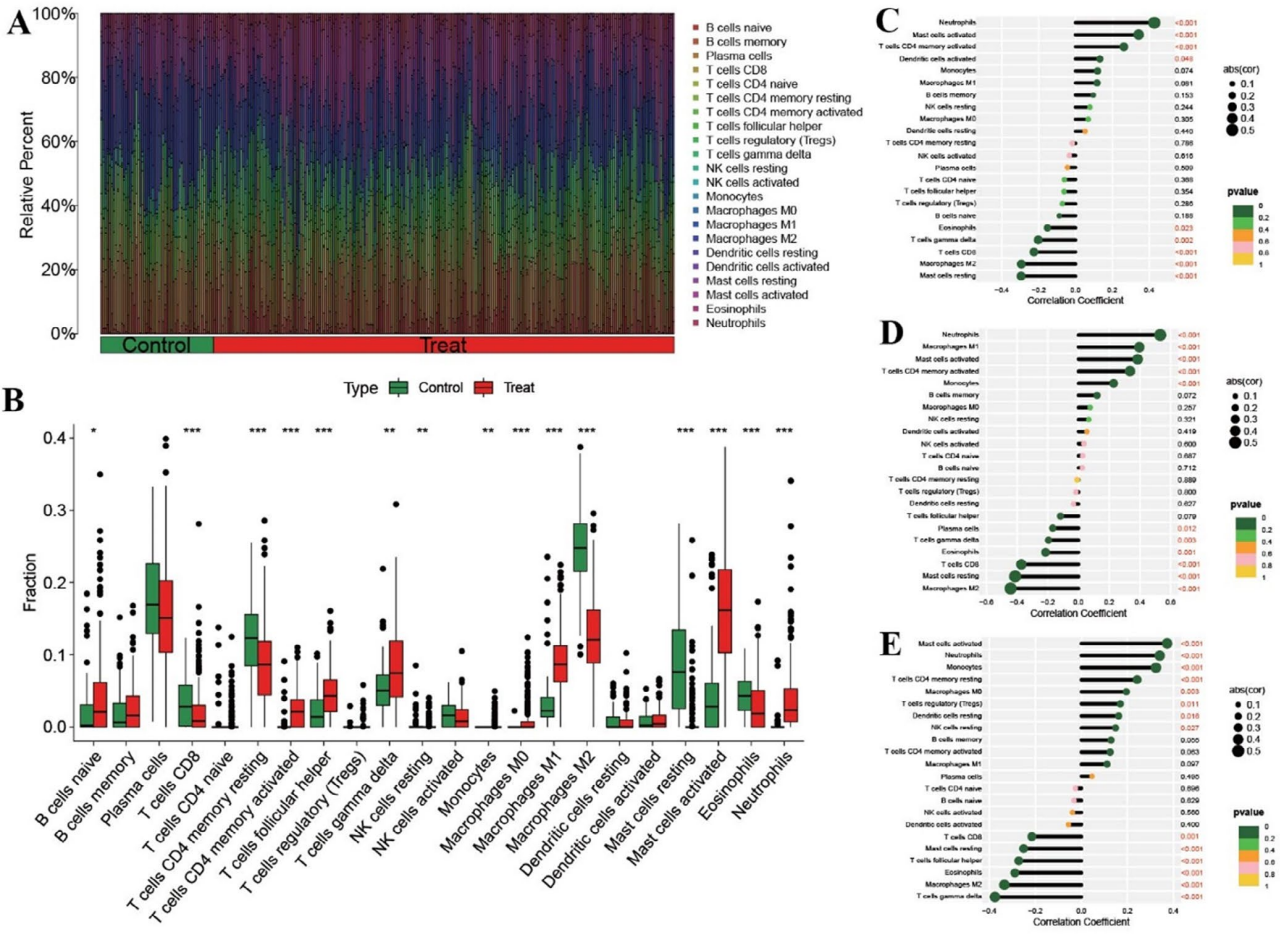
Immune infiltration analysis. (A) The relative percentage of immune cell infiltration of 22 cells in both the control (normal samples) group and treatment (UC samples) group. (B) Violin plots illustrating the differences in immune‐infiltrating cells between control (normal samples) group and treat (UC samples) groups. The horizontal axis represented 22 immune cells, and the vertical axis represented the content of immune cells. *p < 0.05, **p < 0.01, and ***p < 0.001. Infiltration of different immune cells between the disease and normal groups. (C, D and E) Correlation analysis of hub UC‐treated targets (IL17A, JAK2, and STAT3) with immune‐infiltrating cells. The horizontal axis represented a correlation coefficient (positive correlation > 0 and negative correlation < 0), and the vertical axis represented *p* value (*p* < 0.05 indicated significant differences).

The CIBERSORT analysis of immune infiltration abundance revealed a shift in the immune microenvironment between the control group (normal samples) and treatment group (UC samples) (Figure 9A and Figure 9B). Notable variations were observed in the percentage of various immune cells between these two groups (Figure 9B). These immune cells include B cells naïve, T cells CD8, T cells CD4 memory resting, T cells follicular helper, T cells gamma delta, monocytes, macrophages M0, macrophages M1, macrophages M2, eosinophils, and neutrophils. (Figure 9B).

The correlation analysis highlighted that the IL17A expression displayed a positive correlation with neutrophils, mast cells activated, cells CD4 memory activated, and dendritic cells activated while exhibiting a negative correlation with eosinophils, T cells gamma delta, T cells CD8, macrophages M2, and mast cells resting (Figure 9C). Similarly, JAK2 expression was positively correlated with neutrophils, macrophages M1, mast cells activated, cells CD4 memory activated, and monocytes while being negatively correlated with plasma cells, T cells gamma delta, eosinophils, T cells CD8, mast cells resting, and macrophages M2 (Figure 9D). STAT3 expression displayed a positive correlation with mast cells activated, neutrophils, monocytes, T cells CD4 memory resting, macrophages M0, T cells regulatory (Tregs), dendritic cells resting, and NK cells resting while being inversely correlated with T cells CD8, mast cells resting, T cells follicular helper, eosinophils, macrophages M2, and T cells gamma delta (Figure 9E).

### 3.8 Hub UC‐Related Targets Affect Numerous GSEA Pathways and Can Interact With Honokiol and Magnolol

**FIGURE 10:**
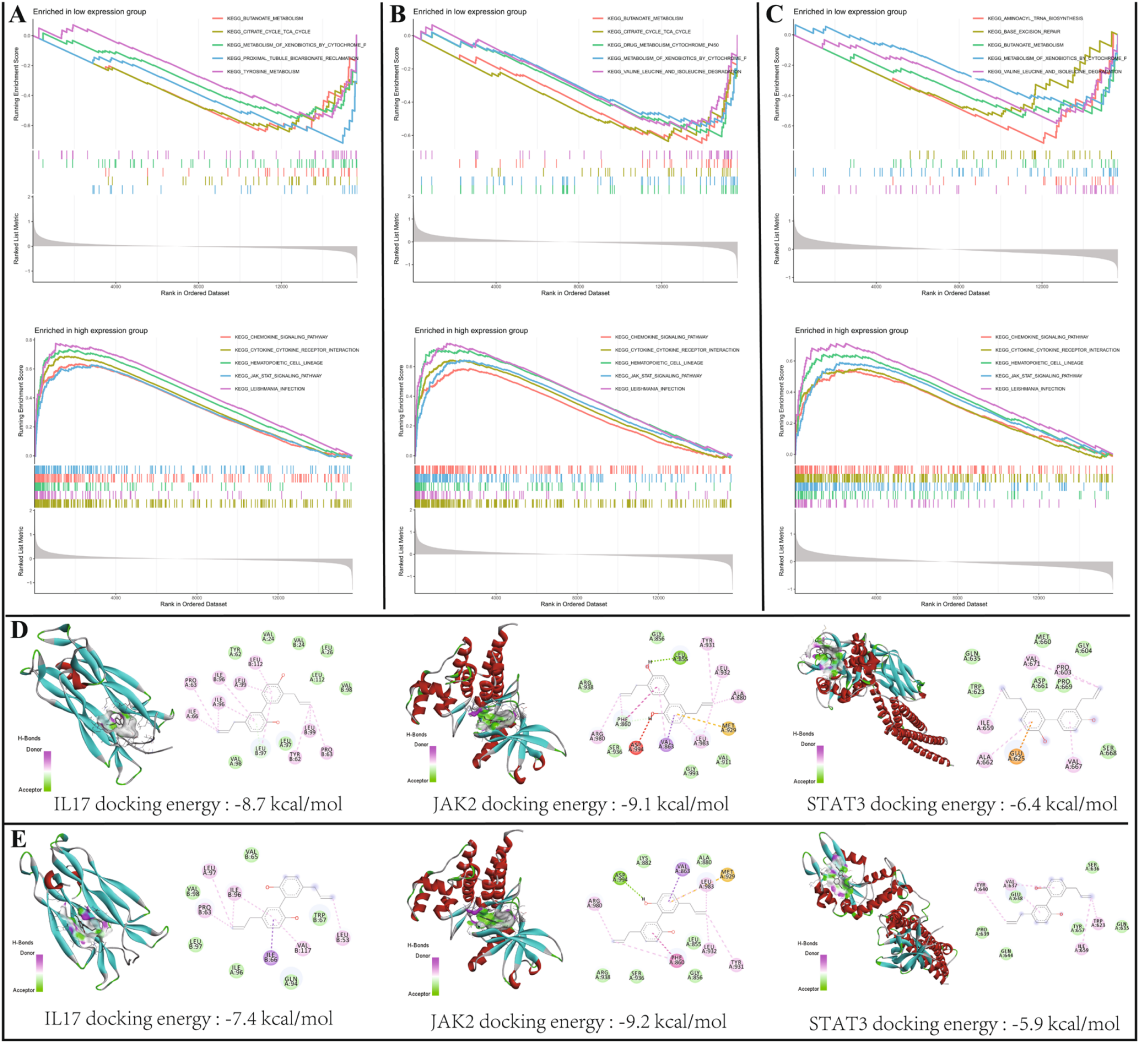
GSEA and molecular docking. (A, B, and C) Top 5 KEGG pathways of IL17A, JAK2, and STAT3 in high‐ and low‐expression groups. (D and E) Molecular docking of hub UC‐treated targets (IL17A, JAK2, and STAT3) with honokiol and magnolol, correspondingly.

GSEA indicated that high expressions of IL17A, JAK2, and STAT3 had a significant association with the activation of JAK–STAT signalling pathway, cytokine–cytokine receptor interaction, Leishmania infection, haematopoietic cell lineage, and chemokine signalling pathway (Figure 10A–10C). Low expression of IL17A affected proximal tubule bicarbonate reclamation, butanoate metabolism, citrate cycle–TCA cycle, metabolism of xenobiotics by cytochrome p450, and tyrosine metabolism (Figure 10A). Low expression of JAK2 imitated drug metabolism cytochrome p450, butanoate metabolism, citrate cycle–TCA cycle, valine, leucine, and isoleucine degradation, and proximal tubule bicarbonate reclamation (Figure 10B), while low expression of STAT3 involved aminoacyl tRNA biosynthesis, valine, leucine, and isoleucine degradation, butanoate metabolism, metabolism of xenobiotics by cytochrome p450, and base excision repair (Figure 10C).

The docking results revealed distinct binding profiles for honokiol and magnolol against IL17A, JAK2, and STAT3 (Figure 10D,E). Honokiol exhibited binding free energies of −8.7, −9.1, and −6.4 kcal/mol with the three targets, respectively, whereas magnolol showed values of −7.4, −9.2, and −5.9 kcal/mol. The stability of each complex was attributed to a network of specific noncovalent interactions, primarily comprising hydrogen bonds and van der Waals forces, as visualised in the docking poses.

### 3.9 Honokiol and Magnolol Might Alleviate UC by Inhibiting the IL17/JAK2/STAT3 Signalling Pathway

**FIGURE 11:**
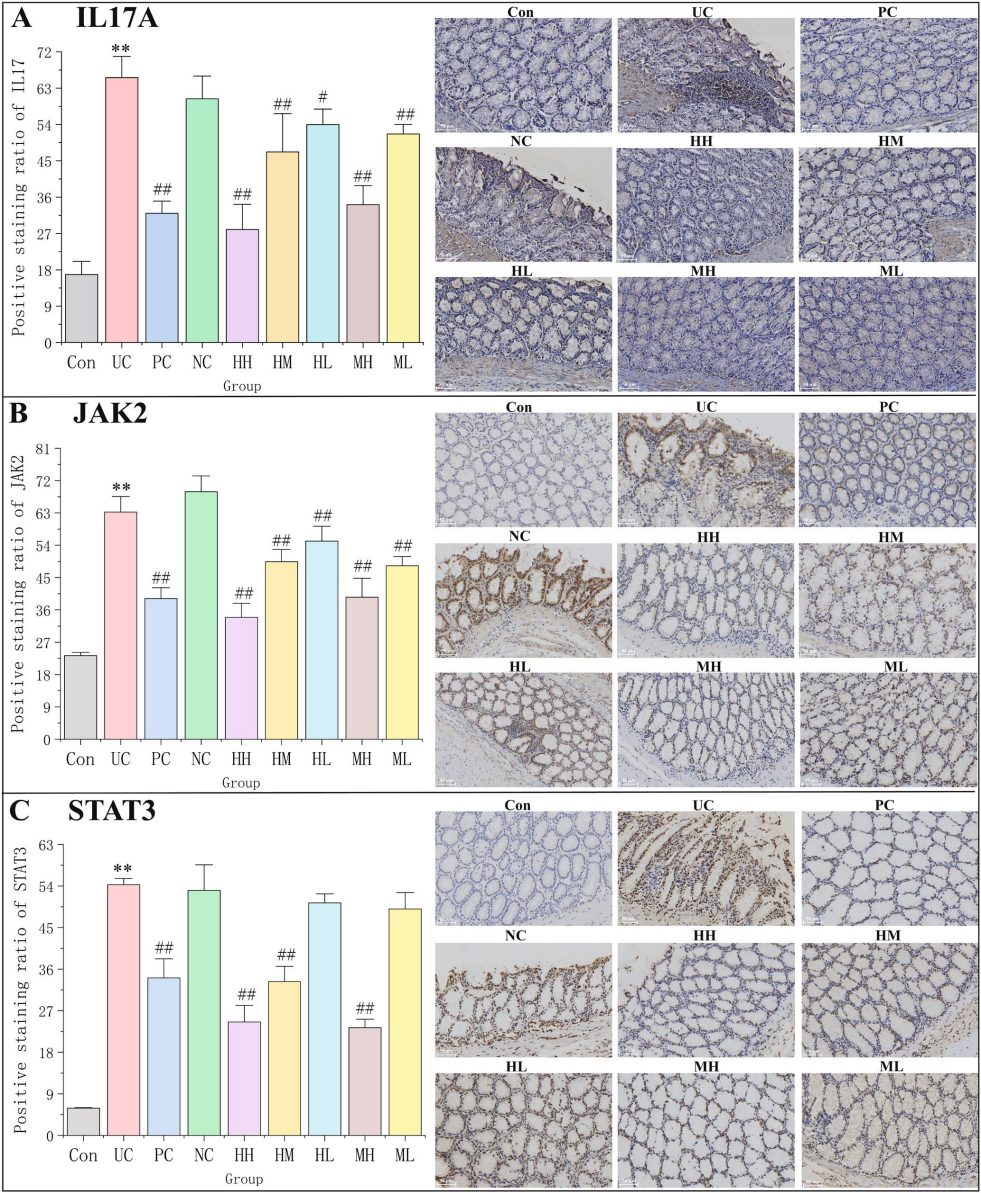
Immunohistochemical analysis. (A, B, and C) Bar plots of staining intensity and immunohistochemical staining (200×) and of IL17, JAK2, and STAT3. Data were shown as the mean ± SD. **p < 0.01 vs. the Con group, ^#^ *p* < 0.05 and ^##^ *p* < 0.01 vs. the UC group.

**FIGURE 12:**
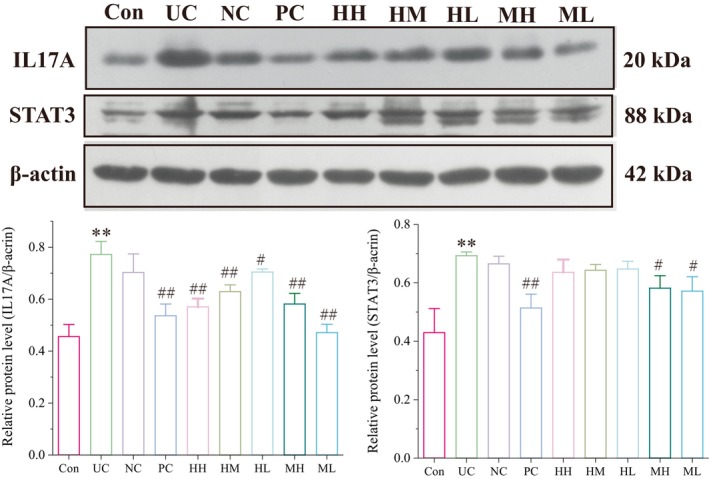
Protein levels of IL17 and STAT3 in rat colonic tissues. Representative images and relative protein levels of IL17 and STAT3 in all groups. Data were represented as mean ± SD. **p < 0.01 vs. the Con group, ^#^ *p* < 0.05 and ^##^ *p* < 0.01 vs. the UC group**.

**FIGURE 13:**
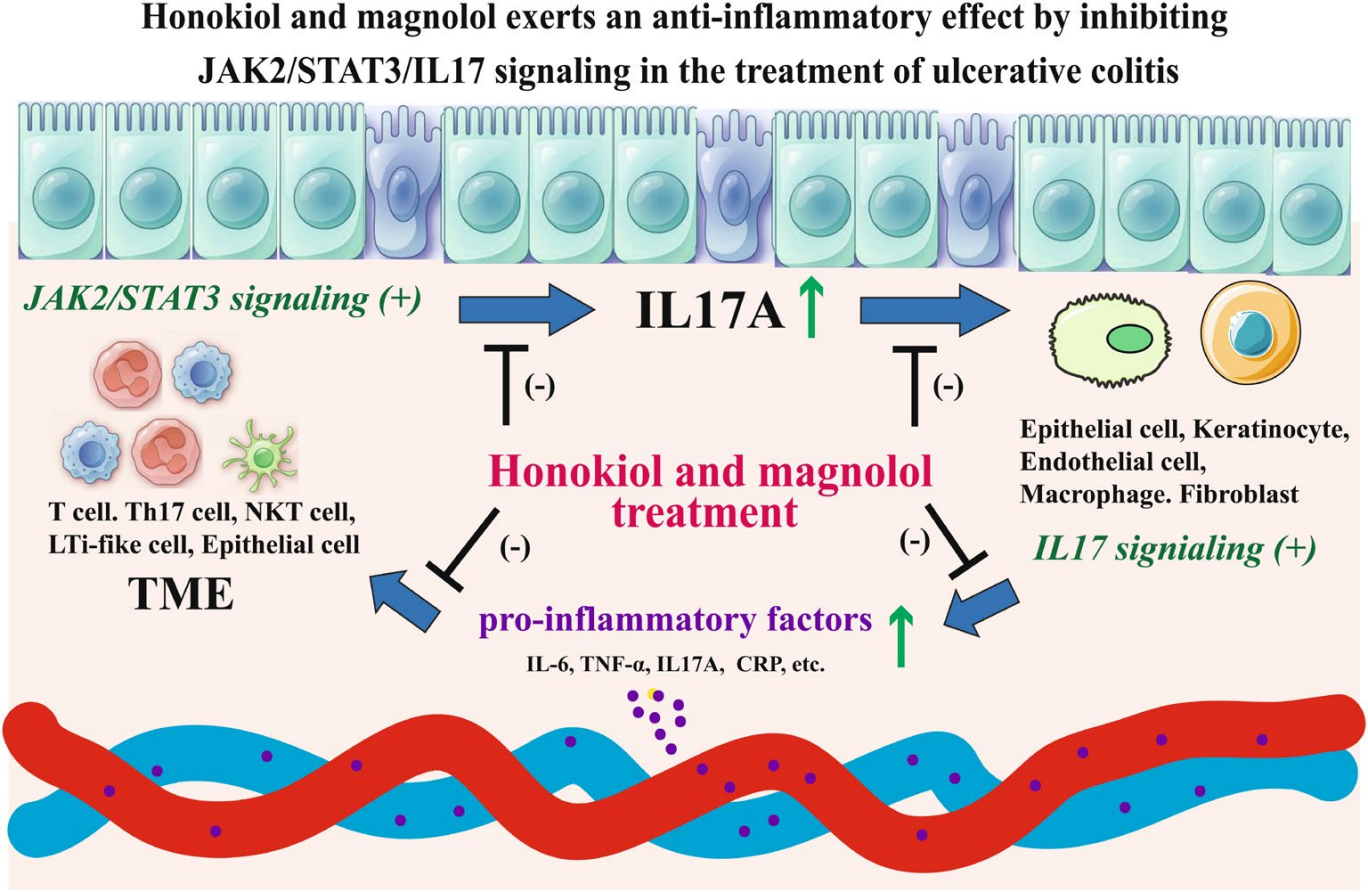
The potential mechanism of honokiol and magnolol in anti‐inflammatory activity of UC.

Based on the above analyses, protein expressions of three hub UC‐treated targets (IL17A, JAK2, and STAT3) in UC rat models were detected using immunohistochemistry and Western blot analysis. Results of immunohistochemistry showed that UC significantly increased the expressions of IL17A, JAK2, and STAT3 in colonic tissues (*p* < 0.01 vs. control). After drug treatment, high protein expression levels of IL17A and JAK2 were markedly reduced in colonic tissues of the HH, HM, HL, MH, and ML groups (Figure 11A,B,p < 0.05 or *p* < 0.01 vs. UC), and STAT3 level was significantly decreased in the HH, HM, and MH groups (Figure 11C,p < 0.01 vs. UC). Consistently, Western blot analysis revealed that the protein expression of IL17A was significantly increased in the UC group (*p* < 0.01 vs. control), and the trend was effectively counteracted by different doses of honokiol and magnolol (*p* < 0.05 or *p* < 0.01 vs. UC) (Figure 12). In addition, UC severely upregulated protein expressions of STAT3 (*p* < 0.01 vs. control), while honokiol treatment revealed a slight shift without significant difference (*p* > 0.05 vs. UC), and magnolol treatment significantly lessened STAT3 level in the context of UC (*p* < 0.05 vs. UC) (Figure 12). In conclusion, the study demonstrated that honokiol and magnolol inhibited the activation of JAK2/STAT3 signalling and repressed the release of proinflammatory factors such as IL‐17A, IL‐6, and TNF‐α, thereby affecting the inflammatory cell infiltration (Figure 13). In addition, the dysregulation of immune cells also affects the tumour microenvironment. IL‐17A might modulate the functions of epithelial cells, keratinocyte, endothelial cells, macrophages, and fibroblasts (Figure 13).

## 4 Discussion

Ulcerative colitis (UC) is a chronic, idiopathic, relapsing inflammatory disease of the gastrointestinal tract [39]. Emerging evidence highlights the therapeutic potential of bioactive compounds derived from traditional Chinese medicine in UC through various mechanisms. For example, luteolin attenuates colonic inflammation and restores gut microbial diversity and composition in a rat model of UC [40]. Quercetin, as the principal active constituent of Xiang‐lian Pill, alleviates experimental colitis in mice by modulating macrophage polarisation through STAT1/PPARγ signalling balance [41]. Similar to these studies, the current study demonstrated that honokiol and magnolol improved colonic mucosal damage and suppressed systemic and local inflammation in a UC rat model by inhibiting the JAK2/STAT3/IL‐17 signalling, as validated by integrated bioinformatic prediction and experimental validation. Notably, the anti‐inflammatory activity of honokiol and magnolol is comparable to that of mesalazine, which is the current first‐line pharmacotherapy for mild‐to‐moderate UC [42]. In addition, treatment of liposomes in the NC group failed to reverse any pathological or biochemical abnormalities in this study, confirming its suitability as a negative control. Consistently, a previous study evidenced that liposomes themselves exhibit no anti‐inflammatory activity and serve solely as inert delivery vehicles for honokiol and magnolol [29]. Moreover, the protective effects of honokiol and magnolol in UC are consistent with observations of recent studies [27, 43, 44, 45]. Differently, prior studies have investigated UC pathogenesis from alternative mechanistic lenses such as pyroptosis, immune cell differentiation, and oxidative stress [27, 44, 45]. The novelty of our study lies in the integrative application of bioinformatics‐guided target prediction and experimental validation, which collectively demonstrate that honokiol and magnolol suppress UC progression by inhibiting the JAK2/STAT3/IL‐17 signalling pathway in vivo.

The rapid advancement of bioinformatics has provided robust computational and analytical frameworks for elucidating complex disease mechanisms, uncovering novel biological functions, and identifying pharmacologically relevant therapeutic targets [46, 47]. To leverage these capabilities, we conducted comprehensive bioinformatics analyses in this study. The criteria for selecting DEGs were |log_2_FC| ≥ 0.5 and adj. *p* < 0.05, which was consistent with previous studies [48, 49, 50]. Our samples exhibited subtle but biologically relevant transcriptomic variations, and many functionally important genes showed relatively small fold changes. Setting a low log_2_FC threshold helps retain biologically meaningful genes with mild expression changes, which are critical for revealing the underlying molecular mechanisms. The adjusted *p*‐value < 0.05 was used to ensure statistical significance and control false positives. Bioinformatics analysis identified JAK2, STAT3, and IL17A as common hub targets shared by honokiol and magnolol in UC, and they were closely related to the infiltration of various immune cells. Given the complexity of the immune microenvironment in UC, further mechanistic studies are warranted to delineate the regulatory effects of honokiol and magnolol on key immune cell populations, including neutrophils, Th17 cells, and M1 macrophages, as well as their intercellular crosstalk within this context.

Clinical studies indicate that elevated serum IL‐17 levels correlate with disease severity and progression in UC ([51]). During Th0‐to‐Th17 differentiation, activation of the JAK2/STAT3 pathway drives the transcriptional upregulation and secretion of inflammatory cytokines, including IL‐17, IL‐21, and IL‐22 [52]. In inflamed colonic tissues, the accumulation of IL‐17 promotes stromal and immune cells to produce pro‐inflammatory factors (e.g., IL‐1β, IL‐6, TNF‐α, and CCL2) and matrix metalloproteinases, ultimately leading to epithelial apoptosis, barrier disruption, and fibrotic remodelling [53, 54]. Integrating these established pathogenic mechanisms with our bioinformatic and experimental findings, we propose a coherent, multi‐layered anti‐inflammatory mechanism for honokiol and magnolol: (1) honokiol and magnolol might inhibit the JAK2/STAT3 pathway during Th17 differentiation, thereby reducing IL‐17A production; (2) attenuation of IL‐17A leads to downregulation of downstream proinflammatory factors, including IL‐6, TNF‐α, and CRP; (3) secondary improvement of the inflammatory immune microenvironment, characterised by reduced infiltration of neutrophils and M1 macrophages and restrained Th17 polarisation, thus establishing a self‐reinforcing therapeutic loop that underlies their potent anti‐inflammatory efficacy.

Beyond the JAK2/STAT3 axis, honokiol and magnolol modulate several additional signalling pathways implicated in UC pathogenesis. For example, magnolol ameliorates dextran sulphate sodium‐induced colitis in mice by lowering NF‐κB activation and enhancing PPAR‐γ signalling, resulting in reduced pro‐inflammatory cytokine production and improved mucosal integrity [43]. Honokiol exerts anti‐colitic effects by inhibiting STAT3/RORγt signalling and thereby suppressing Th17 cell differentiation [27]. Honokiol inhibits gasdermin D‐induced pyroptosis in UC by activating the PPAR‐γ/TLR4/NF‐κB pathway [45] and represses the inflammatory response by inactivating the SLC3A2/L‐leucine/mTORC1/NLRP3 pathway [55]. Collectively, these findings highlight the polypharmacological nature of honokiol and magnolol—targeting convergent inflammatory, metabolic, and cell‐death pathways to achieve synergistic therapeutic effects in UC. Nevertheless, further mechanistic exploration is warranted to identify additional molecular targets and pathway crosstalk, particularly those linking gut barrier integrity, microbiota–host interactions, and tissue‐resident immune reprogramming, which may expand the therapeutic rationale for these compounds.

## 5 Conclusion

Honokiol and magnolol inhibit the activation of the JAK2/STAT3 pathway and suppress downstream IL‐17A secretion in the intestinal immune system, thereby restricting inflammatory cytokines such as IL‐6, TNF‐α, and CRP in colonic tissues and improving the inflammatory microenvironment. The study highlights a novel signalling pathway mediated by honokiol and magnolol in the UC experimental model. Nevertheless, this study has several notable limitations that warrant acknowledgment and future address. First, our investigation was restricted to a single animal model, which may limit the generalizability of our findings to other preclinical models of UC (e.g., trinitrobenzene sulfonic acid‐induced colitis or interleukin‐10 knockout mice) that recapitulate distinct pathological features of human UC. Second, we did not perform mechanistic validation using genetic tools, such as gene knockout mice or siRNA‐mediated target silencing, to directly confirm the causal role of JAK2, STAT3, and IL‐17A in mediating the therapeutic effects of honokiol and magnolol. Third, pharmacokinetic data (e.g., plasma concentration‐time profiles and metabolic transformation of honokiol and magnolol) were not collected, which hinders our understanding of their bioavailability, target engagement efficiency, and potential drug–drug interactions in the context of UC treatment. Fourth, while we explored immune cell infiltration and key signalling pathways, we did not assess the impact of honokiol and magnolol on gut microbiota composition or epithelial barrier function, which are two critical components of UC pathogenesis that may interact with the observed immunomodulatory effects. Moving forward, integrating these missing dimensions with clinical translational studies will be essential to fully elucidate the therapeutic potential and clinical applicability of honokiol and magnolol.

## Author Contributions

**Zhaoxu Cai:** study design and conception, funding acquisition, writing – original draft, writing – review and editing, data curation, formal analysis. **Shaojun Lu:** study design and conception, funding acquisition, writing – original draft, writing – review and editing, data curation, formal analysis. **Jingan Chen:** study design and conception, funding acquisition, writing – original draft, writing – review and editing, data curation, formal analysis. **Xiaoshan Yang:** writing – original draft, writing – review and editing, data curation, formal analysis. **Junlin Lu:** writing – original draft, writing – review and editing, data curation, formal analysis. **Changwen Feng:** funding acquisition, writing – original draft, writing – review and editing, data curation, formal analysis.

## Funding

This work was supported by Guangdong Provincial Bureau of Traditional Chinese Medicine, Chinese Medicine Research Project, 20221457, 20221216. Wu Jieping Medical Research Grant 320.6750.2024‐6‐108, Zhaoqing First Hospital Research Grant YJJ‐2023‐02‐02, YJJ‐2025‐01‐016.

## Ethics Statement

All animal studies received approval from the Animal Ethics Committee at Guangdong Pharmaceutical University (Ethics No: gdpulac2021140).

## Conflicts of Interest

The authors declare no conflicts of interest.

## Data Availability

The data that support the findings of this study are available from the corresponding author upon reasonable request.
